# Rejection for ICU admission: analysis and results of this modality of limitation of therapeutic effort

**DOI:** 10.1186/cc14601

**Published:** 2015-03-16

**Authors:** F Acosta, O Moreno, M Muñoz, R Fernandez, M Colmenero

**Affiliations:** 1 Hospital Universitario San Cecilio, Granada, Spain

## Introduction

The aim was to know the frequency, criteria and implications of rejection for ICU admission to our ICU unit, a secondlevel hospital (18 beds).

## Methods

An observational retrospective study in a time interval of 6 months (January to June 2013). We retrospectively registered all patients rejected for admission to our unit, analyzing the clinical rejection report used in our hospital. From this report we extracted different variables: demographical (age, sex), provenance (emergency room, hospital), clinical (comorbidity, functional situation, diagnosis, reason of requesting admission), rejection motive ('too good', 'too bad', futility, lack of beds, patient rejection), whether it was definitive or conditional, whether the patient was admitted afterwards, and the state at hospital discharge. We realized a descriptive analysis (frequencies) and multivariant analysis of the factors related to futility rejection.

## Results

There were 165 rejections, which represents 25% of total ICU patients evaluated for admission. A total of 59.4% were male. Mean age was 69 ± 7 years (19 to 98). In total, 53.9% had more than two comorbidities (pluripathological) and 31.5% moderate to severe functional disability. The cause of rejection was in 55.2% of situations that the patient was 'too good', 37.6% related to qualitative futility, 4.8% was 'too bad' and in 1.2% a mix of lack of space (beds) and patient rejection. In the multivariant analysis the significant variables related to futility rejection were age (by years) with an OR of 1.05 (1.02 to 1.08), severe functional disability, OR of 4.35 (2.09 to 9.06), and the hospital provenance with an OR of 2.82 (1.1 to 7.2).

## Conclusion

Rejection for admission to ICU units is a frequent medical activity in our day-to-day job. The type of patient most rejected is cardiologic, mostly evaluated for thoracic pain probably ischemic but with low risk. In second place we found patients for which we decide rejection based on subjective qualitative futility, related mostly to age, prior functional disability and provenance.

